# Evidence Supporting Transmission of Severe Acute Respiratory Syndrome Coronavirus 2 While Presymptomatic or Asymptomatic

**DOI:** 10.3201/eid2607.201595

**Published:** 2020-07

**Authors:** Nathan W. Furukawa, John T. Brooks, Jeremy Sobel

**Affiliations:** Centers for Disease Control and Prevention, Atlanta, Georgia, USA

**Keywords:** 2019 novel coronavirus disease, coronavirus disease, COVID-19, severe acute respiratory syndrome coronavirus 2, SARS-CoV-2, viruses, respiratory infections, zoonoses, transmission, asymptomatic, presymptomatic, review

## Abstract

Recent epidemiologic, virologic, and modeling reports support the possibility of severe acute respiratory syndrome coronavirus 2 (SARS-CoV-2) transmission from persons who are presymptomatic (SARS-CoV-2 detected before symptom onset) or asymptomatic (SARS-CoV-2 detected but symptoms never develop). SARS-CoV-2 transmission in the absence of symptoms reinforces the value of measures that prevent the spread of SARS-CoV-2 by infected persons who may not exhibit illness despite being infectious. Critical knowledge gaps include the relative incidence of asymptomatic and symptomatic SARS-CoV-2 infection, the public health interventions that prevent asymptomatic transmission, and the question of whether asymptomatic SARS-CoV-2 infection confers protective immunity.

As the coronavirus disease (COVID-19) pandemic caused by severe acute respiratory syndrome coronavirus 2 (SARS-CoV-2) unfolds, an increasing number of reports have indicated that some infected persons may not exhibit signs or symptoms of illness, including persons who are presymptomatic (SARS-CoV-2 RNA is detectable before symptom onset) or asymptomatic (SARS-CoV-2 RNA is detectable but symptoms never develop) (*1*–*8*). The detection of SARS-CoV-2 RNA in presymptomatic or asymptomatic persons does not prove that they can transmit the virus to others. We describe evidence that supports the concept of transmission while presymptomatic and asymptomatic, which we found during a rapid literature review conducted at the Centers for Disease Control and Prevention (CDC) in early April 2020.

## Evidence Supporting Presymptomatic and Asymptomatic Transmission

We searched the literature in PubMed for articles that were published from January 1 through April 2, 2020, and pertained to presymptomatic or asymptomatic SARS-CoV-2 transmission. This search captured the literature until the time CDC made policy changes recommending community cloth face coverings and universal masking in healthcare facilities. We used combinations of the search terms SARS-CoV-2, COVID-19, asymptomatic, presymptomatic, and transmission. We included original articles, brief reports, and correspondences and excluded reviews, commentaries, opinions, and preprint manuscripts (with the exception of CDC-authored studies that were in review). We classified studies as reporting epidemiologic, virologic, or modeling evidence for presymptomatic or asymptomatic transmission of SARS-CoV-2.

### Epidemiologic Evidence

Most reports of presymptomatic (*9*–*12*), asymptomatic (*13*–*15*), or a combination of presymptomatic or asymptomatic SARS-CoV-2 transmission (*16*,*17*) were from China (Table 1). Presymptomatic or asymptomatic primary patients were typically exposed to SARS-CoV-2 during travel from Wuhan or another city in Hubei Province, China (*9*–*16*). One couple was exposed during a mass gathering in Shanghai for the Chinese Spring Festival (*17*). Reported cases of infected persons who transmitted the virus to others while presymptomatic or asymptomatic have occurred within families or households (*9*–*11*,*13*–*17*), during shared meals (*10*,*12*), or during visits with hospitalized family members (*9*,*13*). An inherent confounder to these reports from China is the inability to entirely rule out alternative SARS-CoV-2 exposure in the community early in the outbreak, when transmission in the community may have been undetected.

**Table 1:** Summary of epidemiologic reports supporting transmission of severe acute respiratory syndrome coronavirus 2 while asymptomatic or presymptomatic*

Ref.	Setting	Primary patient age, y/sex	Primary patient exposure	Transmission type	Time from primary patient exposure to symptoms, d	Secondary patient exposure	Limitations/strengths
(*9*)	Xuzhou, China	56/M	Traveled through Wuhan	Presymptomatic	>5	3 family household members, 3 hospital contacts	L: Possible exposure while visiting a hospital; unclear exposure to the primary patient by the hospital cluster; possible undetected community transmission.
(*10*)	Zhoushan, China	45/M	Lived in Wuhan	Presymptomatic	>3	2 work colleagues sharing dinner	L: Possible exposure from other conference attendees.
(*11*)	Shanghai, China	65/F, 69/M	Lived in Wuhan	Presymptomatic	6	2 family household members	L: Possible undetected community transmission.
(*12*)	Luzhou, China	50/M, 51/F, 23/M	Lived in Wuhan	Presymptomatic	>9	2 family members sharing dinner	L: Possible undetected community transmission.
(*13*)	Anyang, China	20/F	Lived in Wuhan, China	Asymptomatic	NA	5 family household members	L: Initial negative RT-PCR in the primary case; possible undetected community transmission; possible exposure while visiting a hospital.
(*14*)	Nanjing, China	67/M	Traveled to Hubei Province, China	Asymptomatic	NA	3 family household members	L: Possible undetected community transmission.
(*15*)	Beijing, China	48/M	Traveled to Wuhan	Asymptomatic	NA	3 family household members sharing a dinner	L: Possible undetected community transmission.
(*16*)	Guangzhou, China	35/M	Lived in Wuhan	Presymptomatic or asymptomatic	>4	2 family household members	L: Possible infection while the family was in Wuhan; primary patient could have been the wife or son.
(*17*)	Zhejiang, China	58/F, 60/M	Attended Zhejiang Chinese Spring Festival	Presymptomatic or asymptomatic	5	4 family household members	L: Unclear nature of the primary patients’ initial exposure during the visit to a temple; possible undetected community transmission.
(*18*)	Munich, Germany	33/M	Visiting colleague from China was sick	Presymptomatic	3	2 work colleagues	S: The 2 secondary cases had no contact with the sick colleague from China; no community spread in Germany at the time.
(*19*)	Singapore	55/F, 56/M	Visited Wuhan as tourists	Presymptomatic	>4	3 church attendees	S: Limited community spread in Singapore during this time.
	Singapore	54/F	Had dinner with confirmed case-patient	Presymptomatic	11	1 classmate in a singing class	
	Singapore	53/F	Had contact with confirmed case-patient	Presymptomatic	8	1 family household member	
	Singapore	37/M	Traveled to the Philippines	Presymptomatic	>6	1 family household member	
	Singapore	32/M	Traveled to Japan	Presymptomatic	>3	1 household member	
	Singapore	58/F	Had contact with confirmed case-patient	Presymptomatic	5	2 church attendees	
	Singapore	63/M	Traveled to Indonesia	Presymptomatic	>2	1 acquaintance with close contact	

*L, limitation; NA, not applicable; ref., reference; RT-PCR: reverse-transcription PCR; S, strength.

However, cases of presymptomatic transmission have been reported from other countries before widespread community transmission occurred. A report from Germany documented infection of a German businessman after exposure to a mildly symptomatic colleague visiting from China (*18*). Before becoming symptomatic, this businessman exposed 2 other colleagues who subsequently received a COVID-19 diagnosis but did not have contact with the primary patient from China or any other known source. A report from Singapore described 7 COVID-19 clusters resulting from presymptomatic transmission; presymptomatic primary patients varied from persons with travel from high-incidence countries to persons exposed in the local community (*19*). All primary patients experienced distinct periods of initial exposure and presymptomatic close contact with secondary patients who had no other known exposure risks. The incubation periods for presymptomatic primary patients with distinct exposures ranged from 3 to 11 days; for presymptomatic primary patients with travel history to an area with active transmission, the time from last exposure to symptom onset ranged from >2 to >9 days.

### Virologic Evidence

Currently, SARS-CoV-2 infection is primarily diagnosed by detection of viral RNA via reverse transcription PCR (RT-PCR) or by viral culture and demonstration of cytopathic effect (*20*). Although RT-PCR identifies viral RNA and cannot determine whether infectious virus is present, infectiousness can be inferred from cycle threshold (C_t_) values. The RT-PCR C_t_ value represents the number of PCR cycles required to detect SARS-CoV-2 RNA; lower values indicate higher viral load and imply higher infectiousness (*20*–*22*). The exact RT-PCR C_t_ values associated with the presence of infectious SARS-CoV-2 is unknown, but infectious virus has been isolated from a specimen with an RT-PCR C_t_ of 34 (*23*).

Four reports documented the presence of SARS-CoV-2 RNA with lower C_t_ values in samples collected from persons in whom symptoms of COVID-19 never developed (*24*–*27*) (Table 2). Two reports described specimens with low RT-PCR C_t_ values among presymptomatic and asymptomatic residents of a nursing home identified as part of the same outbreak investigation (*23*,*28*). Among these reports, RT-PCR C_t_ values for SARS-CoV-2 RNA in asymptomatically infected persons ranged from 14 to 40 (*23*–*27*). The study with data on presymptomatic infected patients reported an average RT-PCR C_t_ value of 24 (range 15–38) (*23*). Two reports described culture of infectious virus from persons with asymptomatic (*24*) and presymptomatic (*23*) SARS-CoV-2 infection. Although these reports did not identify actual virus transmission while presymptomatic or asymptomatic, the low RT-PCR C_t_ values (i.e., high viral load) and ability to isolate infectious SARS-CoV-2 provide plausible virologic evidence for SARS-CoV-2 transmission by persons not demonstrating symptoms.

**Table 2:** Summary of virologic reports supporting transmission of severe acute respiratory syndrome coronavirus 2 while asymptomatic and presymptomatic*

Reference	Setting	Patient(s), age/sex	Laboratory findings	Limitations
(*23*,*28*)	Nursing home outbreak in Washington	24 presymptomatic and 3 asymptomatic	Mean RT-PCR C_t_ value 24.2 for presymptomatic and 27.3 for asymptomatic patients. Viral culture identified infectious virus in 7 (64%) of 11 specimens from presymptomatic patients; no virus detected in 1 asymptomatic patient.	Incomplete viral culture sampling from all presymptomatic and asymptomatic patients.
(*24*)	Repatriated to Germany from Wuhan, China	2 asymptomatic adults	Patients’ RT-PCR C_t_ values 24 and 30; infectious virus was detected by viral culture for both.	No evidence of transmission during evacuation flight.
(*25*)	Family cluster in Singapore	Asymptomatic 6 mo/M	RT-PCR C_t_ values 14 at diagnosis and increased to 33 over 9 d.	No evidence of transmission from the infant to another household member.
(*26*)	Cluster in Vietnam related to travel to Wuhan	Asymptomatic 55 y/M	RT-PCR C_t_ values >40 at diagnosis and during 9 d of viral RNA shedding.	High C_t_ in the asymptomatic patient suggests minimal infectiousness.
(*27*)	Family cluster in Guangdong, China	Asymptomatic, 26 y/M	RT-PCR C_t_ values 22–32 during testing 7–11 d after initial diagnosis.	No evidence of transmission to other family members in the cluster.

*C_t_, cycle threshold, RT-PCR, reverse transcription PCR.

### Modeling Evidence

Two studies used models to estimate the serial interval (time between symptom onset in a primary patient and the secondary patient) (*29*,*30*) (Table 3). They estimated the serial interval of COVID-19 to be 4 days, which is shorter than the estimated median incubation period for COVID-19 of 5 days (*31*). One report suggested that up to 13% of infections may be transmitted during the presymptomatic period of illness (*29*). These studies relied on reports of primary and secondary cases and may be limited by recall bias; secondary patients are more likely to remember proximal exposures, biasing results toward a shorter serial interval.

**Table 3:** Summary of modeling reports supporting transmission of severe acute respiratory syndrome coronavirus 2 while asymptomatic and presymptomatic*

Reference	Data source	Model findings	Limitations
(*29*)	Confirmed case-patients from 18 provincial health departments in China	The mean serial interval was 4 d, and symptoms developed in 13% of secondary case-patients before primary case-patients, suggesting presymptomatic transmission.	Data limited to reports of confirmed cases early in the outbreak; recall bias may attribute infection to recent exposures and falsely lower the serial interval.
(*30*)	Published articles and case investigation reports.	The median serial interval was 4–5 d, depending on the reports analyzed.	Recall bias may attribute infection to recent exposures and falsely lower the serial interval.
(*32*)	Spatiotemporal data and reports on infections of 375 persons during Spring Festival, China	An estimated 86% of all infections were asymptomatic or mild and not reported; up to 79% of reported cases may have originated from these unreported asymptomatic or mild cases.	Data limited to China early in the outbreak; several assumptions built into a complex model.
(*33*)	Reports of 40 manually selected transmission pairs from China	On the basis of generation times and serial intervals, the authors estimated that one third to one half of transmission occurred from presymptomatic persons.	Data limited for reports of confirmed cases early in the outbreak; recall bias may attribute infection to recent exposures and falsely lower the serial interval.

Two models attempted to estimate the number of infections caused by asymptomatic, presymptomatic, or mildly symptomatic infected persons (*30*,*32*). These models varied widely; 1 model suggested that up to half of infections were transmitted from infected persons who were presymptomatic (*33*), and another suggested that up to four fifths of infections were transmitted by persons with no symptoms or mild symptoms (*32*). Both models suggested that a large number of persons with asymptomatic or mildly symptomatic infections were not detected by the health system and that these persons meaningfully contributed to ongoing community transmission (*32*,*33*). Although models are highly dependent on the assumptions built into them, these models suggest that the speed and extent of SARS-CoV-2 transmission cannot be accounted for solely by transmission from symptomatic persons.

Each of the epidemiologic, virologic, and modeling studies described has limitations. However, in the aggregate, these diverse studies suggest that SARS-CoV-2 can be transmitted by persons with presymptomatic or asymptomatic infection, which may meaningfully contribute to the propagation of the COVID-19 pandemic. This literature summation was conducted to support changes in CDC recommendations to reduce the risk for asymptomatic transmission and was not a systematic review. These conclusions are drawn from the literature available at the time and may change, given the rapidly evolving nature of the evidence base for asymptomatic transmission.

## Public Health Implications of Transmission While Asymptomatic

The existence of persons with asymptomatic SARS-CoV-2 infection who are capable of transmitting the virus to others has several implications. First, the case-fatality rate for COVID-19 may be lower than currently estimated ratios if asymptomatic SARS-CoV-2 infections are included (*34*,*35*). Second, transmission while asymptomatic reinforces the value of community interventions to slow the transmission of COVID-19. Knowing that asymptomatic transmission was a possibility, CDC recommended key interventions including physical distancing (*36*), use of cloth face coverings in public (*37*), and universal masking in healthcare facilities (*38*) to prevent SARS-CoV-2 transmission by asymptomatic and symptomatic persons with SARS-CoV-2 infection. Third, asymptomatic transmission enhances the need to scale up the capacity for widespread testing and thorough contact tracing to detect asymptomatic infections, interrupt undetected transmission chains, and further bend the curve downward.

## Science Questions to Inform Public Health Action

The existence of SARS-CoV-2 transmission while infected persons are presymptomatic and asymptomatic raises 3 key questions that need to be answered to inform public health action. First, the incidence of asymptomatic compared with symptomatic SARS-CoV-2 infection needs to be determined. The extent of presymptomatic or asymptomatic SARS-CoV-2 infection may be clarified by studies using serial virologic data, serologic data, or a combination of both in observational cohorts or surveillance systems. If a substantial proportion of infections are asymptomatic, enhanced testing strategies may be needed to detect these persons. Second, given that a large proportion of infections probably result from transmission from asymptomatic or presymptomatic persons (*32*,*33*,*39*), the effectiveness of public health interventions aimed at reducing their infectiousness needs to be quantified. If the COVID-19 pandemic is found to be driven by undetected asymptomatic or mildly symptomatic SARS-CoV-2 infections, new innovations in disease detection and prevention (beyond exhaustive contact tracing, mass testing, and isolation of asymptomatic contacts) may be needed. Last, knowledge of SARS-CoV-2 immunity among persons with asymptomatic or mild SARS-CoV-2 infection is needed; specifically, whether full or partial immunity develops in these persons, how long protective immunity lasts, and if it is possible to be immune from reinfection but still asymptomatically transmit SARS-CoV-2 while in a carrier state. This information will be crucial for projecting the anticipated course of the pandemic and the potential for SARS-CoV-2 resurgence if immunity wanes (*40*). Information about immunity is also valuable for healthcare and other critical infrastructure workers for whom rates of exposure, and thereby asymptomatic infection, may be higher and who therefore warrant data-informed guidance on how to safely return to work. The answers to these questions will be crucial for guiding the gradual relaxing of community interventions, resuming the normal functions of society, and recovering from the COVID-19 pandemic.

## References

[1] Wang Y, Liu Y, Liu L, Wang X, Luo N, Ling L. Clinical outcome of 55 asymptomatic cases at the time of hospital admission infected with SARS-Coronavirus-2 in Shenzhen, China. J Infect Dis. 2020;jiaa119; Epub ahead of print. 10.1093/infdis/jiaa119 32179910 PMC7184401

[2] Chan JF, Yuan S, Kok KH, To KK, Chu H, Yang J, et al. A familial cluster of pneumonia associated with the 2019 novel coronavirus indicating person-to-person transmission: a study of a family cluster. Lancet. 2020;395:514–23. 10.1016/S0140-6736(20)30154-9 31986261 PMC7159286

[3] Chang L, Zhao L, Gong H, Wang L, Wang L. Severe acute respiratory syndrome coronavirus 2 RNA detected in blood donations. Emerg Infect Dis. 2020;26: Epub ahead of print. 10.3201/eid2607.200839 32243255 PMC7323524

[4] Dong Y, Mo X, Hu Y, Qi X, Jiang F, Jiang Z, et al. Epidemiological characteristics of 2143 pediatric patients with 2019 coronavirus disease in China. Pediatrics. 2020; [Epub ahead of print]. 10.1542/peds.2020-0702

[5] Mizumoto K, Kagaya K, Zarebski A, Chowell G. Estimating the asymptomatic proportion of coronavirus disease 2019 (COVID-19) cases on board the Diamond Princess cruise ship, Yokohama, Japan, 2020. Euro Surveill. 2020;25:25; Epub ahead of print. 10.2807/1560-7917.ES.2020.25.10.2000180 32183930 PMC7078829

[6] Roxby AC, Greninger AL, Hatfield KM, Lynch JB, Dellit TH, James A, et al. Detection of SARS-CoV-2 among residents and staff members of an independent and assisted living community for older adults– Seattle, Washington, 2020. MMWR Morb Mortal Wkly Rep. 2020;69:416–8. 10.15585/mmwr.mm6914e2 32271726 PMC7147909

[7] Tian S, Hu N, Lou J, Chen K, Kang X, Xiang Z, et al. Characteristics of COVID-19 infection in Beijing. J Infect. 2020;80:401–6. 10.1016/j.jinf.2020.02.018 32112886 PMC7102527

[8] Ng O-T, Marimuthu K, Chia P-Y, Koh V, Chiew CJ, De Wang L, et al. SARS-CoV-2 infection among travelers returning from Wuhan, China. N Engl J Med. 2020;382:1476–8. 10.1056/NEJMc2003100 32163698 PMC7121487

[9] Li C, Ji F, Wang L, Wang L, Hao J, Dai M, et al. Asymptomatic and human-to-human transmission of SARS-CoV-2 in a 2-family cluster, Xuzhou, China. Emerg Infect Dis. 2020 Mar 31 [Epub ahead of print]. 10.3201/eid2607.200718 PMC7323514 32228809

[10] Tong ZD, Tang A, Li KF, Li P, Wang HL, Yi JP, et al. Potential presymptomatic transmission of SARS-CoV-2, Zhejiang Province, China, 2020. Emerg Infect Dis. 2020;26:1052–4. 10.3201/eid2605.200198 32091386 PMC7181913

[11] Yu P, Zhu J, Zhang Z, Han Y, Huang L. A familial cluster of infection associated with the 2019 novel coronavirus indicating potential person-to-person transmission during the incubation period. J Infect Dis. 2020;jiaa077; Epub ahead of print. 10.1093/infdis/jiaa077 32067043 PMC7107453

[12] Ye F, Xu S, Rong Z, Xu R, Liu X, Deng P, et al. Delivery of infection from asymptomatic carriers of COVID-19 in a familial cluster. Int J Infect Dis. 2020;94:133–8; Epub ahead of print. 10.1016/j.ijid.2020.03.042 32247826 PMC7129961

[13] Bai Y, Yao L, Wei T, Tian F, Jin DY, Chen L, et al. Presumed asymptomatic carrier transmission of COVID-19. JAMA. 2020;323:1406. 10.1001/jama.2020.2565 32083643 PMC7042844

[14] Hu Z, Song C, Xu C, Jin G, Chen Y, Xu X, et al. Clinical characteristics of 24 asymptomatic infections with COVID-19 screened among close contacts in Nanjing, China. Sci China Life Sci. 2020;63:706–11. 10.1007/s11427-020-1661-4 32146694 PMC7088568

[15] Zhang J, Tian S, Lou J, Chen Y. Familial cluster of COVID-19 infection from an asymptomatic. Crit Care. 2020;24:119. 10.1186/s13054-020-2817-7 32220236 PMC7100442

[16] Pan X, Chen D, Xia Y, Wu X, Li T, Ou X, et al. Asymptomatic cases in a family cluster with SARS-CoV-2 infection. Lancet Infect Dis. 2020;20:410–1. 10.1016/S1473-3099(20)30114-6 32087116 PMC7158985

[17] Qian G, Yang N, Ma AHY, Wang L, Li G, Chen X, et al. A COVID-19 Transmission within a family cluster by presymptomatic infectors in China. Clin Infect Dis. 2020;ciaa316; Epub ahead of print. 10.1093/cid/ciaa316 32201889 PMC7184331

[18] Rothe C, Schunk M, Sothmann P, Bretzel G, Froeschl G, Wallrauch C, et al. Transmission of 2019-nCoV infection from an asymptomatic contact in Germany. N Engl J Med. 2020;382:970–1. 10.1056/NEJMc2001468 32003551 PMC7120970

[19] Wei WE, Li Z, Chiew CJ, Yong SE, Toh MP, Lee VJ. Presymptomatic Transmission of SARS-CoV-2 - Singapore, January 23-March 16, 2020. MMWR Morb Mortal Wkly Rep. 2020;69:411–5. 10.15585/mmwr.mm6914e1 32271722 PMC7147908

[20] Loeffelholz MJ, Tang YW. Laboratory diagnosis of emerging human coronavirus infections - the state of the art. Emerg Microbes Infect. 2020;9:747–56. 10.1080/22221751.2020.1745095 32196430 PMC7172701

[21] Corman VM, Landt O, Kaiser M, Molenkamp R, Meijer A, Chu DKW, et al. Detection of 2019 novel coronavirus (2019-nCoV) by real-time RT-PCR. Euro Surveill. 2020;25:25; Epub ahead of print. 10.2807/1560-7917.ES.2020.25.3.2000045 31992387 PMC6988269

[22] Wölfel R, Corman VM, Guggemos W, Seilmaier M, Zange S, Müller MA, et al. Virological assessment of hospitalized patients with COVID-2019. Nature. 2020; Epub ahead of print. 10.1038/s41586-020-2196-x 32235945

[23] Arons MM, Hatfield KM, Reddy SC, Kimball A, James A, Jacobs JR, et al. Presymptomatic SARS-CoV-2 infections and transmission in a skilled nursing facility. N Engl J Med. 2020;NEJMoa2008457; Epub ahead of print. 10.1056/NEJMoa2008457 32329971 PMC7200056

[24] Hoehl S, Rabenau H, Berger A, Kortenbusch M, Cinatl J, Bojkova D, et al. Evidence of SARS-CoV-2 infection in returning travelers from Wuhan, China. N Engl J Med. 2020;382:1278–80. 10.1056/NEJMc2001899 32069388 PMC7121749

[25] Kam KQ, Yung CF, Cui L, Lin Tzer Pin R, Mak TM, Maiwald M, et al. A well infant with coronavirus disease 2019 with high viral load. Clin Infect Dis. 2020;ciaa201; Epub ahead of print. 10.1093/cid/ciaa201 32112082 PMC7358675

[26] Le TQM, Takemura T, Moi ML, Nabeshima T, Nguyen LKH, Hoang VMP, et al. Severe acute respiratory syndrome coronavirus 2 shedding by travelers, Vietnam, 2020. Emerg Infect Dis. 2020;26: Epub ahead of print. 10.3201/eid2607.200591 32240079 PMC7323563

[27] Zou L, Ruan F, Huang M, Liang L, Huang H, Hong Z, et al. SARS-CoV-2 viral load in upper respiratory specimens of infected patients. N Engl J Med. 2020;382:1177–9. 10.1056/NEJMc2001737 32074444 PMC7121626

[28] Kimball A, Hatfield KM, Arons M, James A, Taylor J, Spicer K, et al.; Public Health – Seattle & King County; CDC COVID-19 Investigation Team. Public Health – Seattle & King County; CDC COVID-19 Investigation Team. Asymptomatic and presymptomatic SARS-CoV-2 infections in residents of a long-term care skilled nursing facility—King County, Washington, March 2020. MMWR Morb Mortal Wkly Rep. 2020;69:377–81; Epub ahead of print. 10.15585/mmwr.mm6913e1 32240128 PMC7119514

[29] Du Z, Xu X, Wu Y, Wang L, Cowling BJ, Meyers LA. Serial interval of COVID-19 among publicly reported confirmed cases. Emerg Infect Dis. 2020;26: Epub ahead of print. 10.3201/eid2606.200357 32191173 PMC7258488

[30] Nishiura H, Linton NM, Akhmetzhanov AR. Serial interval of novel coronavirus (COVID-19) infections. Int J Infect Dis. 2020;93:284–6. 10.1016/j.ijid.2020.02.060 32145466 PMC7128842

[31] Lauer SA, Grantz KH, Bi Q, Jones FK, Zheng Q, Meredith HR, et al. The incubation period of coronavirus disease 2019 (COVID-19) from publicly reported confirmed cases: estimation and application. Ann Intern Med. 2020; Epub ahead of print. 10.7326/M20-0504 32150748 PMC7081172

[32] Li R, Pei S, Chen B, Song Y, Zhang T, Yang W, et al. Substantial undocumented infection facilitates the rapid dissemination of novel coronavirus (SARS-CoV-2). Science. 2020;368:489–93; Epub ahead of print. 10.1126/science.abb3221 32179701 PMC7164387

[33] Ferretti L, Wymant C, Kendall M, Zhao L, Nurtay A, Abeler-Dörner L, et al. Quantifying SARS-CoV-2 transmission suggests epidemic control with digital contact tracing. Science. 2020;eabb6936; Epub ahead of print. 10.1126/science.abb6936 PMC7164555 32234805

[34] Verity R, Okell LC, Dorigatti I, Winskill P, Whittaker C, Imai N, et al. Estimates of the severity of coronavirus disease 2019: a model-based analysis. Lancet Infect Dis. 2020;S1473-3099(20)30243-7; Epub ahead of print. 10.1016/S1473-3099(20)30243-7 32240634 PMC7158570

[35] Wu JT, Leung K, Bushman M, Kishore N, Niehus R, de Salazar PM, et al. Estimating clinical severity of COVID-19 from the transmission dynamics in Wuhan, China. Nat Med. 2020;26:506–10. 10.1038/s41591-020-0822-7 32284616 PMC7094929

[36] Lasry A, Kidder D, Hast M, Poovey J, Sunshine G, Winglee K, et al. Timing of community mitigation and changes in reported covid-19 and community mobility—four US metropolitan areas, February 26–April 1, 2020. MMWR Morb Mortal Wkly Rep. 2020 Apr 13 [Epub ahead of print]. 10.15585/mmwr.mm6915e2 PMC7755061 32298245

[37] Mahase E. Covid-19: What is the evidence for cloth masks? BMJ. 2020;369:m1422. 10.1136/bmj.m1422 32265341

[38] Klompas M, Morris CA, Sinclair J, Pearson M, Shenoy ES. Universal masking in hospitals in the COVID-19 era. N Engl J Med. 2020;NEJMp2006372; Epub ahead of print. 10.1056/NEJMp2006372 32237672

[39] He X, Lau EHY, Wu P, Deng X, Wang J, Hao X, et al. Temporal dynamics in viral shedding and transmissibility of COVID-19. Nat Med. 2020; Epub ahead of print. 10.1038/s41591-020-0869-5 32296168

[40] Kissler SM, Tedijanto C, Goldstein E, Grad YH, Lipsitch M. Projecting the transmission dynamics of SARS-CoV-2 through the postpandemic period. Science. 2020;eabb5793; Epub ahead of print. 10.1126/science.abb5793 PMC7164482 32291278

